# Nonlinear Behavior of Electrostatically Actuated Microbeams with Coupled Longitudinal–Transversal Vibration

**DOI:** 10.3390/mi10050315

**Published:** 2019-05-10

**Authors:** Chicheng Ma, Limin Cao, Lei Li, Mingyu Shao, Dong Jing, Zonghe Guo

**Affiliations:** School of Transportation and Vehicle Engineering, Shandong University of Technology, Zibo 255000, China; limincao1024@163.com (L.C.); shaomingyu@sdut.edu.cn (M.S.); jingrenzhi@163.com (D.J.); guozonghe@sdut.edu.cn (Z.G.)

**Keywords:** electrostatically actuated microbeam, coupled longitudinal–transversal vibration, pseudo-arclength continuation method, multiple-scales method

## Abstract

Microelectromechanical switch has become an essential component in a wide variety of applications, ranging from biomechanics and aerospace engineering to consumer electronics. Electrostatically actuated microbeams and microplates are chief parts of many MEMS instruments. In this study, the nonlinear characteristics of coupled longitudinal–transversal vibration are analyzed, while an electrostatically actuated microbeam is designed considering that the frequency ratio is two to one between the first longitudinal vibration and transversal vibration. The nonlinear governing equations are truncated into a set of coupled ordinary differential equations by the Galerkin method. Then the equations are solved using the multiple-scales method and the nonlinear dynamics of the internal resonance is investigated. The influence of bias voltage, longitudinal excitation and frequency detuning parameters are mainly analyzed. Results show that using the pseudo-arclength continuation method, the nonlinear amplitude–response curves can be plotted continuously. The saturation and jump phenomena are greatly affected by the bias voltage and the detuning frequency. Beyond the critical excitation amplitude, the response energy will transfer from the longitudinal motion to the transversal motion, even the excitation is employed on the longitudinal direction. The large-amplitude jump of the low-order vibration mode can be used to detect the variation of the conditions or parameters, which shows great potential in improving precision of MEMS switches.

## 1. Introduction

Electrostatic actuation is the most popular actuation mechanism used in microelectromechanical systems (MEMS) [1,2]. Various electrostatic switches and resonators have been used in a wide variety of applications, such as in biomechanics, aerospace engineering, and consumer electronics. Understanding the mechanical behavior of microbeams [3,4,5,6] and microplates [7,8,9] is of great importance due to their applications in devices such as in resonators, sensors, and actuators [10,11], and nonlinear vibration analysis of micro/nanosystems has been widely studied based on continuum theories [12,13,14,15,16,17].

Electrostatically actuated microbeams and plates exhibit significant nonlinearities due to the action of the electrostatic force, as the electrostatic force is inversely proportional to the square of the distance between the electrode and the host structure. On account of nonlinearities, a collapse of the movable structure occurs at a critical voltage (pull-in instability), and the phenomenon can be used as change of ON or OFF state [18,19,20,21,22,23,24,25]. Younis [26,27] used analytical approaches to investigate the behavior of electrically actuated microbeam-based MEMS, and proposed a reduced-order model in nonlinear dynamic analysis of MEMS. Alsaleem [28] showed experimental and theoretical investigations of dynamic pull-in of electrostatically actuated resonators, and the influences of different conditions were investigated. Ouakad and Younis [29,30] studied the dynamic behavior of clamped–clamped micromachined arches with a Galerkin-based reduced-order model, a variety of nonlinear phenomena was analyzed in detail, such as hysteresis, softening behavior, dynamic snap-through, and dynamic pull-in. Wang [31] presented a size-dependent model for electrostatically actuated microbeam-based MEMS using strain gradient elasticity theory, in the study the relation of the geometry size and the normalized pull-in voltage were analyzed in detail.

Early studies mainly focused on the static and dynamic behavior of microbeam-based MEMS. Li [32] gave monostable dynamic analysis of microbeam-based resonators using an improved reduced-order model, results showed that the ratio of the gap width to microbeam thickness affected large-amplitude vibration significantly. Ghayesh [33] investigated the nonlinear size-dependent behavior of an electrically actuated MEMS resonator based on the modified couple stress theory and a high-dimensional reduced-order model. Based on reduced-order model, Caruntu [34] researched parametric resonance of microelectromechanical (MEMS) cantilever resonators under soft damping and soft alternating current (AC) electrostatic actuation. Kacem [35] constructed a comprehensive multiphysics model for electrostatically actuated clamped–clamped resonators based on the Galerkin decomposition method coupled with the averaging method. Feng [36] showed detailed analysis of class of bipolar electrostatically actuated micro-resonators, and the electrostatic force nonlinearity, neutral surface tension, and neutral surface bending were considered. Han [37] investigated the static and dynamic characteristics of a doubly clamped micro-beam-based resonator driven by two electrodes, nonlinear dynamic analysis for two cases were analyzed, including that the origin of the system was at a stable center or an unstable saddle point.

To get more precise results, Muldavin [38] presented an accurate model of the switching mechanism of MEMS switches based on an electro-mechanical analysis, varying force and damping versus position (time) were taken into consideration. Ghayesh [39] investigated the size-dependent dynamical performance of a microgyroscope using the modified couple stress theory, and size-dependent frequency–response curves for the sense and drive directions were obtained to analyze the dynamical performance of the microgyroscope. Li [40] used the modified couple stress-based strain gradient theory to construct a unified nonlinear model for an electrostatic MEMS microbeam capacitive switch, the quasistatic and dynamic behavior of which were studied systematically. Leus [41] applied energy methods to study the undamped dynamic response of electrostatic MEMS switches under a step-function voltage. Larose [42] analyzed the influences of inertial effects, structural, air damping (squeeze-film damping) and the impact behavior of the microbeam. Lin [43] mainly investigated the action of Casimir effect on the pull-in parameters of nanometer switches. Shear deformation was taken into account in the analysis of microbeams with geometric nonlinearities [44,45,46,47,48,49].

The dynamical behavior of microelectromechanical systems (MEMS) plays a pivotal role in determining their performance, such as natural frequency [50,51,52,53], critical pull-in voltage [54,55,56], bifurcations [57,58]. Dynamical bifurcations have been taken into account in MEMS sensors for mass detection [59,60,61], which may lead to new mass measurement architectures to miniaturized mass spectrometers [62]. Studies on coupled vibrations have introduced a host of nonlinear phenomena in micro- and nano-scale resonators for their use to reveal the mechanism of the complex dynamic behaviors. Alkharabsheh [63] investigated the effect of axial forces on the static behavior and the fundamental natural frequency of electrostatically actuated MEMS arches. Li [64] investigated the nonlinear characteristics of microbeam-based resonators under higher-order modes excitation.

It can be concluded that coupled vibration behaviors caused by internal resonance are gradually considered, and the jump phenomenon is also focused on [65,66,67]. The phenomenon is interesting, but the application of this phenomenon is scarce. Classical MEMS switches are usually designed based on pull-in instability, here this study aims to investigate application of jump phenomena in nonlinear bifurcation in improving the precision of MEMS switches. A nonlinear model for a microbeam is established, taking electrostatic force, axial load, coupled longitudinal–transversal vibrations and mid-plane stretching into consideration. A single mode approximation is derived and the physical condition for the jump is determined, then the influence of longitudinal excitation and frequency detuning parameters are analyzed qualitatively. Pseudo-arclength continuation method and a direct time-integration technique are used to investigate the nonlinear dynamic behavior of the electrostatically actuated microbeam due to the axial load. Using numerical method, frequency-amplitude responses and force-amplitude responses are given to demonstrate the nonlinear characteristics.

The rest of the paper is arranged as follows: In Section 2, mathematical model is given using Galerkin’s method, and the multiple-scales method is used to derive an approximate average equation. Static analysis with convergence analysis and perturbation analysis are shown in Section 3 and Section 4, respectively. A brief introduction of pseudo-arclength continuation is given in Section 5. Section 6 gives resonant conditions for this kind of electrostatically actuated microbeam. In Section 7, nonlinear dynamical properties of the resonant microbeam are investigated in detail. Several conclusions are summarized in the last section.

## 2. Mathematical Model

**Figure 1:**
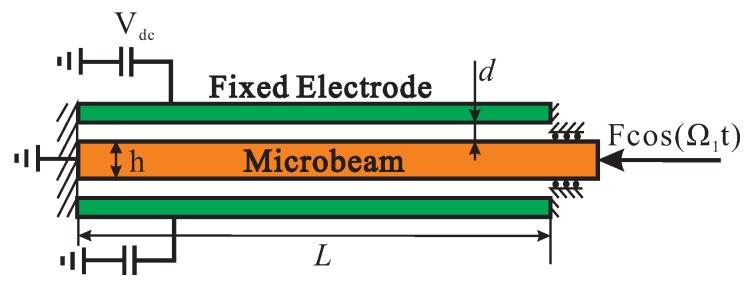
Schematic of an electrically actuated microbeam.

A slender microbeam under the action of electric field and axial load is considered, as shown in Figure 1. The total length of the beam is *L*, with a thickness of *h* and width of *b*. The distance between the fixed electrode and the microbeam is *d*. On the right end of the beam, an axial force is applied. Two electric field $V_{dc}$ is applied to detune the stiffness of the switch. In the following derivation, same assumptions from Reference [33] are to be applied:(1) the Euler-Bernoulli beam theory is applied without regard to shear deformation effect and rotary inertia; (2) the microbeam and the parallel-plate electrodes have a complete overlapping area; (3) the geometric nonlinearity is caused by the mid-plane stretching of the microbeam; (4) the transversal deflection is constant along the width.

By Hamilton’s principle and nonlinear Euler-Bernoulli beam theory, the coupled longitudinal–transversal vibration of the microbeam can be written as [32,52]:

(1) $$\rho A\frac{\partial^{2}\bar{u}}{\partial t^{2}}+c_{1}\frac{\partial \bar{u}}{\partial t}-EA\frac{\partial^{2}\bar{u}}{\partial x^{2}}-EA\;\frac{\partial \bar{w}}{\partial x}\frac{\partial^{2}\bar{w}}{\partial x^{2}}={\bar{F}}_{1}\cos\left(\Omega_{1}t\right)$$

(2) $$\begin{array}{c} \rho A\frac{\partial^{2}\bar{w}}{\partial t^{2}}+c_{2}\frac{\partial \bar{w}}{\partial t}+EI_{z}\frac{\partial^{4}\bar{w}}{\partial x^{4}}=EA\left(\frac{\partial^{2}\bar{u}}{\partial x^{2}}\frac{\partial \bar{w}}{\partial x}+\frac{\partial \bar{u}}{\partial x}\frac{\partial^{2}\bar{w}}{\partial x^{2}}\right)\\ +\frac{EA}{2L}\int_{0}^{L}{\left(\frac{\partial \bar{w}}{\partial x}\right)}^{2}dx\frac{\partial^{2}\bar{w}}{\partial x^{2}}+\frac{1}{2}\frac{\epsilon_{0}bCV_{dc}^{2}}{{\left(d-\bar{w}\right)}^{2}}-\frac{1}{2}\frac{\epsilon_{0}bCV_{dc}^{2}}{{\left(d+\bar{w}\right)}^{2}} \end{array}$$

where $\bar{u}$ is the longitudinal displacement and $\bar{w}$ is the transversal displacement; *E* and $I_{z}$ are the Young’s modulus and moment of inertia of the cross section of the microbeam, ρ is the material density and *A* is the area of the cross section. An axial force $\bar{F}\cos\left(\Omega_{1}t\right)$ is applied at the right end of the microbeam. The terms on the right-hand side of Equation (2) represent the axial force, mid-plane stretching effects, and two parallel-plate electric actuation, respectively. In the approximated electric forces, *C* is the fringing field coefficient and $\epsilon_{0}$ is the dielectric constant of the gap medium. It should be mentioned that the fringing field always exists in the electrostatically actuated microbeams. However, in this study, we aim to investigate the bifurcation phenomena and its potential in improving the precision of MEMS switches. Considering that the response trend will not be influenced by the fringing effect, the fringing effect is neglected for the sake of simplicity, and here C=1.

The boundary conditions for this microbeam are:

(3) $$\bar{w}\left(0,t\right)=0,{\bar{w}}^{\prime }\left(0,t\right),\bar{w}\left(L,t\right)=0,{\bar{w}}^{\prime }\left(L,t\right),\bar{u}\left(0,t\right)=0,\bar{u}\left(L,t\right)=0$$

To simplify the dynamic equations, the following dimensionless variables are applied:

(4) $$x=\frac{\bar{x}}{L},w=\frac{\bar{w}}{g},u=\frac{\bar{u}}{g},t=\frac{\bar{t}}{\tau }$$

where $\tau =\sqrt{\frac{\rho AL^{4}}{EI_{z}}}$ is the time scale.

Substituting nondimensional variables into Equations (1) and (2) yields

(5) $$\frac{\partial^{2}u}{\partial t^{2}}+\mu_{1}\frac{\partial u}{\partial t}-\mu_{2}\frac{\partial^{2}u}{\partial x^{2}}-\mu_{2}\frac{g}{L}\;\frac{\partial w}{\partial x}\frac{\partial^{2}w}{\partial x^{2}}=F_{1}\cos\left(\Omega t\right)$$

(6) $$\begin{array}{c} \frac{\partial^{2}w}{\partial t^{2}}+\mu_{1}\frac{\partial w}{\partial t}+\frac{\partial^{4}w}{\partial x^{4}}=\nu_{1}\left(\frac{\partial^{2}u}{\partial x^{2}}\frac{\partial w}{\partial x}+\frac{\partial u}{\partial x}\frac{\partial^{2}w}{\partial x^{2}}\right)\\ +\nu_{2}\int_{0}^{1}{\left(\frac{\partial w}{\partial x}\right)}^{2}dx\frac{\partial^{2}w}{\partial x^{2}}+\nu_{3}\left(\frac{V^{2}}{{\left(1-w\right)}^{2}}-\frac{V^{2}}{{\left(1+w\right)}^{2}}\right) \end{array}$$

where the new parameters are given as:

(7) $$\begin{array}{c} \mu_{1}=\frac{cL^{4}}{\tau EI_{z}},\mu_{2}=\frac{AL^{2}}{I_{z}},F_{1}={\bar{F}}_{1}\frac{L^{4}}{EI_{z}}\\ \Omega =\Omega_{1}\tau ,\nu_{1}=\frac{dAL}{I_{z}},\nu_{2}=6\frac{d^{2}}{h^{2}},\nu_{3}=\frac{6\epsilon_{0}L^{4}}{Ed^{3}h^{3}} \end{array}$$

To analyze the dynamic equations qualitatively, a reduced-order model is firstly derived using Galerkin’s method, transforming the complex equations into a multi-degree of freedom system. The solutions of the equations can be presented by mode shapes and generalized coordinates:

(8) $$w\left(x,t\right)=\sum_{k=1}^{m}\phi_{k}\left(x\right)q_{k}\left(t\right),u\left(x,t\right)=\sum_{k=1}^{n}\psi_{k}\left(x\right)p_{k}\left(t\right)$$

where $\phi_{k}\left(x\right)$ and $\psi_{k}\left(x\right)$ are mode shapes for transversal vibration and longitudinal vibration, respectively; $q_{k}\left(t\right)$ and $p_{k}\left(t\right)$ are generalized coordinates for transversal vibration and longitudinal vibration, respectively. The linear undamped mode shape of the transversal vibration of the straight beam is:

(9) $$\phi_{k}=A_{k}\left(\cos\beta_{k}x-\cosh\beta_{k}x+\frac{\cosh\beta_{k}-\cos\beta_{k}}{\sin\beta_{k}-\sinh\beta_{k}}\left(\sin\beta_{k}x-\sinh\beta_{k}x\right)\right)$$

where $\lambda_{k}=\beta_{k}^{2}\sqrt{\frac{EI_{z}}{\rho A}}$, and the following relationship should also be satisfied for $\beta_{k}$:

(10) $$1-\cos\beta_{k}x\cosh\beta_{k}x=0$$

For the transversal vibration of the straight beam, one characteristic of the linear undamped mode shape is:

(11) $$\frac{\partial^{4}\phi_{i}\left(x,t\right)}{\partial x^{4}}=\lambda_{i}^{2}\phi_{i}\left(x\right),\phi_{i}\left(0\right)=\phi_{i}\left(1\right)=\phi_{i}^{\prime }\left(0\right)=\phi_{i}^{\prime }\left(1\right)=0$$

For longitudinal vibration of a fixed-free beam, the linear undamped mode shape has the form:

(12) $$\psi_{k}=B_{k}\sin\alpha_{k}x$$

where $\alpha_{k}=\frac{(2k+1)\pi }{2L}\sqrt{\frac{E}{\rho }}$ and $\beta_{k}L=\left(4.7300,8.8532,10.9956,\cdots \right)$.

In Galerkin’s method, the linear mode shapes of the beam can be used as basis functions, and the differential Equation (5) have the following equivalent form:

(13) $$\begin{array}{c} \frac{\partial^{2}p}{\partial t^{2}}\int_{0}^{1}\psi \psi dx+\mu_{1}\frac{\partial p}{\partial t}\int_{0}^{1}\psi \psi dx-\mu_{2}p\int_{0}^{1}\psi \psi dx\\ -\mu_{2}\frac{g}{L}q^{2}\int_{0}^{1}\phi_{x}\phi_{xx}\psi dx=F_{1}\psi \left(L\right)\cos\left(\Omega t\right) \end{array}$$

For small *w*, the electric actuation force is simplified using Taylor approximation, and carried out as follows [68]:

(14) $$\frac{V^{2}}{{\left(1-w\right)}^{2}}-\frac{V^{2}}{{\left(1+w\right)}^{2}}=\frac{4wV^{2}}{{\left[(1+w)(1-w)\right]}^{2}}\approx 4wV^{2}\left(1+2w^{2}\right)$$

Using Equations (11) and (14), multiplying Equation (6) by ϕ, and integrating the outcome from x=0 to 1, yields:

(15) $$\begin{array}{c} \frac{\partial^{2}q}{\partial t^{2}}\int_{0}^{1}\phi \phi dx+\mu_{1}\frac{\partial q}{\partial t}\int_{0}^{1}\phi \phi dx+q\int_{0}^{1}\phi_{xxxx}\phi dx-\nu_{1}pq\int_{0}^{1}\left(\psi_{xx}\phi_{x}+\psi_{x}\phi_{xx}\right)\phi dx=\\ \nu_{2}q^{3}\int_{0}^{1}\int_{0}^{1}\phi_{x}^{2}dx\phi_{xx}\phi dx+4\nu_{3}V^{2}q\int_{0}^{1}\phi \phi dx+8\nu_{3}V^{2}q^{3}\int_{0}^{1}\phi^{3}\phi dx=0 \end{array}$$

Taking the orthogonality of mode shapes and Equation (11) into consideration, and only use the first order mode function, the coupling transversal-longitudinal vibration equations are obtained:

(16) $$\ddot{p}+\mu_{1}\dot{p}-\mu_{2}p-\gamma_{1}q^{2}=F_{1}\cos\left(\Omega t\right)$$

(17) $$\ddot{q}+\mu_{1}\dot{q}+\lambda^{2}q-\gamma_{2}pq-\gamma_{3}q-\gamma_{4}q^{3}=0$$

where

(18) $$\begin{array}{c} F_{1}=F_{1}\psi \left(L\right)\\ \gamma_{1}=\mu_{2}\frac{g}{L}\int_{0}^{1}\phi_{x}\phi_{xx}\psi dx\\ \gamma_{2}=\nu_{1}\int_{0}^{1}\left(\psi_{xx}\phi_{x}\phi +\psi_{x}\phi_{xx}\phi \right)dx\\ \gamma_{3}=4\nu_{3}V^{2}\\ \gamma_{4}=\nu_{2}\int_{0}^{1}\int_{0}^{1}\phi_{x}^{2}dx\phi_{xx}\phi dx+8\nu_{3}V^{2}\int_{0}^{1}\phi^{3}\phi dx \end{array}$$

## 3. Static Analysis and Convergence Analysis

**Figure 2:**
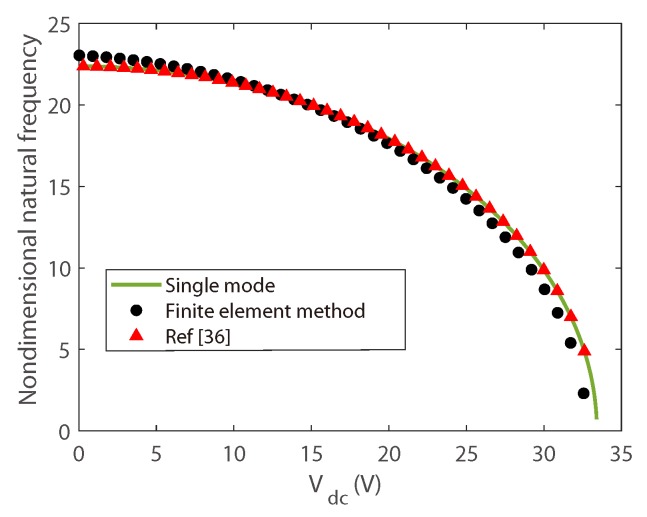
Comparison of eigenfrequency of the first transversal vibration.

An example of eigenfrequency analysis in the steady state are given at first. Here, results by single mode Galerkin approximation, finite element simulations, and results from previous literature are performed. The parameters from previous literature is applied in the calculation, and the calculated simulation results are shown in Figure 2, one can see that the results agree well with each other.

**Figure 3:**
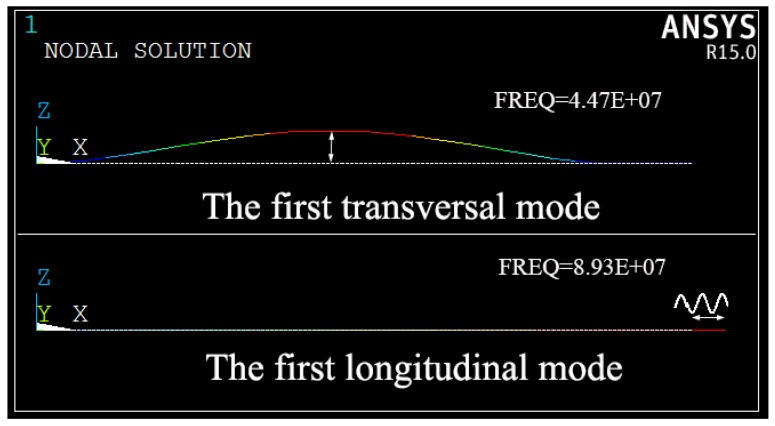
The first transversal mode shape and longitudinal mode shape.

To activate the internal resonance between two vibrational modes, dimensions of the switch need carefully designed. A two-to-one frequency ratio between the longitudinal mode and transversal mode can cause the energy exchange between the two modes. However, a perfectly 2:1 frequency ratio is not always effective and feasible in practical engineering, as small changes in structural dimensions and material properties can deviate the nominal 2:1 ratio. In the numerical simulation, the material and geometric parameters for the microbeam are designed as *E* = 169 GPa, ρ = 2300 kg/m${}^{3}$, *L* = 24 μm, *h* = 2.92 μm, *g* = 0.5 μm and *b* = 10 μm. Using finite element software ANSYS R15.0, modal analysis is carried out, and the first transversal mode shape and longitudinal mode shape are plotted in Figure 3. The natural frequency of the first longitudinal mode is 8.93 × 10${}^{7}$ Hz and the natural frequency of the first transversal mode is 4.47 × 10${}^{7}$ Hz, therefore a frequency ratio near two to one is obtained. Then a bias DC voltage between the electrodes and microbeam is applied, resulting in two electrostatic forces which will change the mechanical stiffness of the microbeam and consequently a frequency ratio of 2:1 is ensured.

**Figure 4:**
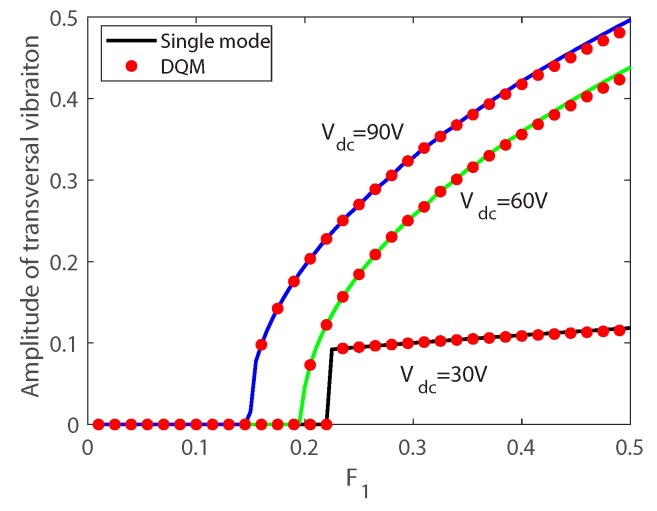
Comparison of the force-amplitude response curves obtained by single mode Galerkin approximation and differential quadrature method.

To verify the validity of the coupled Equations (16) and (17), the convergence analysis is carried out, comparing the results obtained by the reduced-order model and the results by direct numerical simulation of Equations (5) and (6). The direct numerical simulation is given by differential quadrature method [32]. The force-amplitude curves are simulated as shown in Figure 4, and three cases with different bias voltages are calculated. The results predicted by those two methods agree very well for small deformations. Therefore, the single mode model can describe the dynamic behavior of the system with small deformation assumption. It is interesting that two kinds of force-amplitude responses are obtained, one for $V_{dc}$ = 30 V with a jump in the amplitude response, and another for $V_{dc}$ = 60 V and $V_{dc}$ = 90 V with continually increasing amplitude, and the difference will be explained in Section 7.

## 4. Perturbation Analysis

In this section, the method of multiple-scales is to be applied to analyze the steady-state responses of the microbeam around equilibrium position. Before the analysis, a small nondimensional bookkeeping parameter ϵ is introduced to indicate the significance of each terms in Equations (16) and (17). Taking the electrostatic force term $F_{1}=O\left(\epsilon^{2}\right)$ into consideration, the coupling vibration equations become:

(19) $$\begin{array}{c} \ddot{p}+\epsilon^{2}\mu_{1}\dot{p}+\mu_{2}p+\gamma_{1}q^{2}=\epsilon^{2}F_{1}\cos\left(\Omega t\right) \end{array}$$

(20) $$\begin{array}{c} \ddot{q}+\epsilon^{3}\mu_{1}\dot{q}+\lambda^{2}q+\gamma_{2}pq+\gamma_{3}q+\gamma_{4}q^{3}=0 \end{array}$$

From Equation (19), one can find that there is no transversal vibration when the amplitude of external force $F_{1}$ is small or driving frequency Ω is far away from two times of the first nature frequency of the transversal vibration. For the two-to-one internal resonance, two parameters Δ and δ are introduced to describe the nearby vicinity of the resonance.

(21) $$\Omega =\omega_{p}-\epsilon^{2}\delta ,\omega_{p}=2\omega_{q}-\epsilon^{2}\Delta$$

Based on multiple-scales method, the approximate solutions of the longitudinal vibration and transversal vibration can be defined in the following forms:

(22) $$\begin{array}{c} p=\epsilon p_{1}\left(T_{0},T_{1},T_{2}\right)+\epsilon^{2}p_{2}\left(T_{0},T_{1},T_{2}\right)+\epsilon^{3}p_{3}\left(T_{0},T_{1},T_{2}\right)+\cdots \end{array}$$

(23) $$\begin{array}{c} q=\epsilon q_{1}\left(T_{0},T_{1},T_{2}\right)+\epsilon^{2}q_{2}\left(T_{0},T_{1},T_{2}\right)+\epsilon^{3}p_{3}\left(T_{0},T_{1},T_{2}\right)+\cdots \end{array}$$

where $T_{n}=\epsilon^{n}t$ represents different timescale.

Substituting the approximate solutions into the dynamic equations and equating the coefficients of equal powers of ϵ, a series of second-order ordinary differential equations will be obtained.

For $O(\epsilon^{1})$:

(24) $$D_{0}^{2}p_{1}+\omega_{p}^{2}p_{1}=0,D_{0}^{2}q_{1}+\omega_{q}^{2}q_{1}=0$$

For $O(\epsilon^{2})$:

(25) $$D_{0}^{2}p_{2}+w_{p}^{2}p_{2}=\gamma_{1}p_{1}^{2}-2D_{0}D_{1}p_{1}+F_{1}\cos\left(\Omega T_{0}\right)$$

(26) $$D_{0}^{2}q_{2}+w_{q}^{2}q_{2}=\gamma_{2}p_{1}q_{1}-2D_{0}D_{1}q_{1}$$

For $O(\epsilon^{3})$:

(27) $$D_{0}^{2}p_{3}+w_{p}^{2}p_{3}=-\left(D_{1}^{2}+2D_{0}D_{2}\right)p_{1}-2D_{0}D_{1}p_{2}-\mu_{1}D_{0}p_{1}+2\gamma_{1}p_{1}p_{2}$$

(28) $$D_{0}^{2}q_{3}+w_{p}^{2}q_{3}=-\left(D_{1}^{2}+2D_{0}D_{2}\right)q_{1}-2D_{0}D_{1}q_{2}+2\gamma_{2}\left(p_{1}q_{2}+p_{2}q_{1}\right)-\gamma_{4}q_{1}^{3}$$

where $\omega_{p}^{2}=\mu_{2}$ and $\omega_{q}^{2}=\lambda^{2}-\gamma_{3}$, and the differential operator $D_{n}$ indicates the derivative with respect to timescale $T_{n}$.

The general solutions of Equation (24) can be written as

(29) $$\begin{array}{c} p_{1}=A_{1}\left(T_{1},T_{2}\right)e^{j\omega_{p}T_{0}}+{\bar{A}}_{1}\left(T_{1},T_{2}\right)e^{-j\omega_{p}T_{0}}\\ q_{1}=B_{1}\left(T_{1},T_{2}\right)e^{j\omega_{q}T_{0}}+{\bar{B}}_{1}\left(T_{1},T_{2}\right)e^{-j\omega_{q}T_{0}} \end{array}$$

where $\bar{A}$ and $\bar{B}$ are complex conjugate functions of *A* and *B*, and *j* represents imaginary component. For convenience, the parameters in above solutions can be represented in polar form:

(30) $$A_{1}=\frac{1}{2}a_{1}e^{j\theta_{p}},B_{1}=\frac{1}{2}b_{1}e^{j\theta_{q}}$$

where $a_{1}$ and $b_{1}$ represent the amplitudes of the first longitudinal vibration and the first transversal vibration, respectively.

Substituting Equations (29) and (30) into Equations (25)–(28), the following secular terms are yielded:

(31) $${\dot{a}}_{1}=\frac{\gamma_{1}a_{1}b_{1}}{4\omega_{q}}\sin\left(\varphi \right)-\frac{\mu_{1}b_{1}}{2}$$

(32) $$\dot{\varphi }=\delta +\Delta +\frac{\gamma_{1}a_{1}}{2w_{q}}\cos\left(\varphi \right)+k_{1}b_{1}^{2}+k_{2}a_{1}^{2}$$

(33) $${\dot{b}}_{1}=-\frac{\gamma_{1}b_{1}^{2}}{4\omega_{p}}\sin\left(\varphi \right)-\frac{\mu_{1}a_{1}}{2}-\frac{F_{1}}{2\omega_{p}}\sin\left(\chi \right)$$

(34) $$\dot{\chi }=\delta +\frac{\gamma_{3}b_{1}^{2}}{4\omega_{p}a_{1}}\cos\left(\varphi \right)+k_{3}b_{1}^{2}-\frac{F_{1}}{2\omega_{p}a_{1}}\cos\left(\chi \right)$$

The papameters φ, χ, $k_{1}$, $k_{2}$ and $k_{3}$ are defined as:

(35) $$\varphi =2\theta_{q}+\Delta t-\theta_{p},\chi =\delta t+\theta_{p}$$

(36) $$k_{1}=\frac{3\gamma_{2}}{4\omega_{q}}-\frac{\gamma_{1}\gamma_{3}}{2\omega_{p}^{2}\omega_{q}}$$

(37) $$k_{2}=\frac{\gamma_{1}^{2}}{32\omega_{q}^{3}-24\omega_{q}^{2}\Delta }$$

(38) $$k_{3}=\frac{\gamma_{1}\gamma_{3}}{32\omega_{q}^{2}\omega_{p}-24\omega_{q}\omega_{p}\Delta }$$

To obtain the steady-state solutions, one can set all the time derivatives $\left({\dot{a}}_{1},\dot{\varphi },{\dot{b}}_{1},\dot{\chi }\right)$ to be zero and all the time-dependent variables to be constants in Equations (31)–(34), then solve the algebraic equations with the right-hand side zero. The stability of steady-state solutions can be determined by the eigenvalues of Jacobian matrix of Equations (31)–(34) near the steady-state solutions. If all the eigenvalues of the Jacobian matrix have non-positive real parts, the system is stable otherwise the system is unstable. Finally, the steady-state frequency responses are determined by the following frequency-response equations:

(39) $$c_{n}^{2}+s^{2}-\frac{\gamma_{1}^{2}a_{1}^{2}}{4\omega_{2}^{2}}=0$$

(40) $${\left(\frac{2\gamma_{3}b_{1}^{2}\omega_{2}}{\gamma_{1}a_{1}}+\frac{c_{n}a_{1}}{2}\right)}^{2}+{\left[\left(\delta +k_{3}b_{1}^{2}\right)a_{1}-\frac{\gamma_{3}b_{1}^{2}\omega_{2}}{2\gamma_{1}a_{1}\omega_{3}}s^{2}\right]}^{2}=\frac{F_{1}^{2}}{4\omega_{3}^{2}}$$

where $s=\delta +\Delta +k_{2}a_{1}^{2}+k_{1}b_{1}^{2}$.

With the Routh-Hurwitz criterion, stability of the system can be easily analyzed by the characteristic polynomial. Routh-Hurwitz stability criterion supplies a set of necessary and sufficient conditions for the accurate delineation of the relevant parameter space into stable and unstable regions. To determine the stability of the periodic solution, the Jacobian matrix of Equations (31)–(34) is obtained as shown in Equations (31)–(41), then the stability of the solutions is analyzed by the Routh-Hurwitz criterion.

(41) $$J=\left[\begin{array}{cccc} \frac{\gamma_{1}b_{1}}{4\omega_{q}}\sin\left(\varphi \right) & \frac{\gamma_{1}a_{1}b_{1}}{4\omega_{q}}\cos\left(\varphi \right) & \frac{\gamma_{1}a_{1}}{4\omega_{q}}\sin\left(\varphi \right)-\frac{\mu_{1}}{2} & 0\\ \frac{\gamma_{1}}{2w_{q}}\cos\left(\varphi \right)+2k_{2}a_{1} & -\frac{\gamma_{1}a_{1}}{2w_{q}}\sin\left(\varphi \right) & 2k_{1}b_{1} & 0\\ -\frac{\mu_{1}}{2} & -\frac{\gamma_{1}b_{1}^{2}}{4\omega_{p}}\cos\left(\varphi \right) & -\frac{2\gamma_{1}b_{1}}{4\omega_{p}}\sin\left(\varphi \right) & -\frac{F_{1}}{2\omega_{p}}\cos\left(\chi \right)\\ -\frac{\gamma_{3}b_{1}^{2}}{4\omega_{p}a_{1}^{2}}\cos\left(\varphi \right)+\frac{F_{1}}{2\omega_{p}a_{1}^{2}}\cos\left(\chi \right) & -\frac{\gamma_{3}b_{1}^{2}}{4\omega_{p}a_{1}}\sin\left(\varphi \right) & \frac{\gamma_{3}b_{1}}{2\omega_{p}a_{1}}\cos\left(\varphi \right)+2k_{3}b_{1} & \frac{F_{1}}{2\omega_{p}a_{1}}\sin\left(\chi \right) \end{array}\right]\;$$

## 5. Pseudo-Arclength Continuation

To analyze the steady states, Equations (39) and (40) are calculated by a two-step pseudo-arclength continuation method. Firstly, one advances along a branch of steady states with a varied parameter, then the linear stability analysis of the most recently computed steady state is carried out [69,70].

Equations (39) and (40) can be written in the matrix form:

(42) Φ(u,ς)=0

where $u=(a_{1},b_{1})$ and ς is the variation parameter. Function Φ(u,p) contains two functions here:

(43) $$\Phi_{1}=c_{n}^{2}+s^{2}-\frac{\gamma_{1}^{2}a_{1}^{2}}{4\omega_{2}^{2}}$$

(44) $$\Phi_{2}={\left(\frac{2\gamma_{3}b_{1}^{2}\omega_{2}}{\gamma_{1}a_{1}}+\frac{c_{n}a_{1}}{2}\right)}^{2}+{\left[\left(\delta +k_{3}a_{2}^{2}\right)a_{1}-\frac{\gamma_{3}b_{1}^{2}\omega_{2}}{2\gamma_{1}a_{1}\omega_{3}}s^{2}\right]}^{2}-\frac{F_{1}^{2}}{4\omega_{3}^{2}}$$

The pseudo-arclength continuation is naturally a predictor-corrector method, parametrizing branches of solutions Γ(s)=(u(s),ς(s)) with an arclength parameter *s*. For every given solution $\left(u_{0},\varsigma_{0}\right)$ and the next solution (u,ς), the following relationship should be satisfied for a small step size Δs:

(45) $${\dot{u}}_{0}^{T}\left(u-u_{0}\right)+{\dot{\varsigma }}_{0}\left(\varsigma -\varsigma_{0}\right)-\Delta s=0$$

Furthermore, a normalization equation is supplemented to solve the extended system [70]:

(46) $$|{\dot{\Gamma }}_{0}\left(s\right)|=1$$

where ${\dot{\Gamma }}_{0}=\left({\dot{u}}_{0},{\dot{\varsigma }}_{0}\right)$ represents the normalized direction vector of the solution family Γ(s) at $\left(u_{0},\varsigma_{0}\right)$.

To compute the normalized direction vector $\Gamma_{0}$, one can solve the following equation:

(47) $$\left[\Phi_{u}^{0},\Phi_{\varsigma }^{0}\right]{\dot{\Gamma }}_{0}=0$$

where $\Phi_{u}$ is the derivative of Jacobian matrix to parameter u and $\Phi_{\varsigma }$ is the derivative of Jacobian matrix to parameter ς at point $\left(u_{0},\varsigma_{0}\right)$. The predictor solution of is given by

(48) $$u^{0}=u_{0}+\Delta s{\dot{u}}_{0},\varsigma^{0}=\varsigma_{0}+\Delta s{\dot{\varsigma }}_{0}$$

In corrector algorithm, the predictor solution $\left(u_{0},\varsigma_{0}\right)$ is projected back to the branch in a direction orthogonal to the tangent ${\dot{\Gamma }}_{0}$. The Newton-Raphson iterations is used here, and the iteration is performed as:

(49) $$\left[\begin{array}{cc} \Phi_{u}\left(u^{k},\varsigma^{k}\right) & \Phi_{\varsigma }\left(u^{k},\varsigma^{k}\right)\\ {\dot{u}}_{0}^{T} & {\dot{\varsigma }}_{0} \end{array}\right]\;\left[\begin{array}{c} \Delta u^{k+1}\\ \Delta \varsigma^{k+1} \end{array}\right]\;=\left[\begin{array}{c} -\Phi (u^{k},\varsigma^{k})\\ r^{k} \end{array}\right]\;$$

where $r^{k}=\Delta s-{\dot{u}}_{0}^{T}\left(u^{k}-u_{0}\right)-{\dot{\varsigma }}_{0}\left(\varsigma^{k}-\varsigma_{0}\right)$. After getting the new iteration direction, $\left(u^{k},\varsigma^{k}\right)$ is updated by $\left(u^{k+1},\varsigma^{k+1}\right)$.

(50) $$u^{k+1}=u^{k}+\Delta u^{k+1}$$

(51) $$\varsigma^{k+1}=\varsigma^{k}+\Delta \varsigma^{k+1}$$

In practical calculation, it is sometimes better to solve two n×n linear systems instead of directly solving, namely

(52) $$\Phi_{u}\left(u^{k},\varsigma^{k}\right)z_{1}=-\Phi \left(u^{k},\varsigma^{k}\right)$$

(53) $$\Phi_{u}\left(u^{k},\varsigma^{k}\right)z_{2}=\Phi_{\varsigma }\left(u^{k},\varsigma^{k}\right)$$

Finally, the new iteration direction can be obtained using the new parameters $z_{1}$ and $z_{2}$:

(54) $$\Delta \varsigma^{k+1}=\frac{r^{k}-{\dot{u}}_{0}^{T}z_{1}}{{\dot{\varsigma }}_{0}-{\dot{u}}_{0}^{T}z_{1}}$$

(55) $$\Delta u^{k+1}=z_{1}+\Delta \varsigma^{k+1}z_{2}$$

The advantage of pseudo-arclength method is that the Jacobian matrix of the extended system has rank *n* [69], even at folds where $\Phi_{u}$ becomes singular. Hence, using pseudo-arclength method, one can plot the frequency responses curves easily without piecewise process, and the stable and unstable solutions around folds can be computed continuously.

## 6. The Resonant Condition

The problem of Equations (1) and (2) can be classified as a parametrically excited system. The resonance occurs if the excitation frequency of the longitudinal vibration approaches twice of the any frequency of the transversal vibration, and the resonance curve typically displays a trivial solution [58]. To determine the critical state of the dynamic system, it is advantageous to transform the general solutions from polar coordinates $b_{1}$ and φ to rectangular coordinates η and ϱ:

(56) $$\eta =b_{1}\cos\frac{\varphi }{2},\varrho =b_{1}\sin\frac{\varphi }{2}$$

Substituting Equation (56) into Equations (31) and (32), the derivatives of η and ϱ can be obtained:

(57) $$\dot{\eta }=-\frac{c_{n}}{2}\eta +\left(\frac{\gamma_{1}a_{1}}{4\omega_{q}}-\frac{\delta +\Delta +k_{2}a_{1}^{2}}{2}\right)\varrho -\frac{k_{1}}{2}\left(\eta^{2}+\upsilon^{2}\right)\varrho$$

(58) $$\dot{\varrho }=-\frac{c_{n}}{2}\varrho +\left(\frac{\gamma_{1}a_{1}}{4\omega_{q}}+\frac{\delta +\Delta +k_{2}a_{1}^{2}}{2}\right)\eta +\frac{k_{1}}{2}\left(\eta^{2}+\upsilon^{2}\right)\eta$$

Similar to above introductions, the stability of steady-state solutions of Equations (57) and (58) can also be determined by the eigenvalues of the linearized coefficients matrix (i.e., Jacobian matrix) near the steady-state solutions. If all the eigenvalues of the Jacobian matrix all have negative real part at an equilibrium point, the point is asymptotically stable, the system is stable, otherwise at least one eigenvalue has positive real part, the equilibrium is unstable. The linearized coefficients matrix has the following form:

(59) $$J=\left[\begin{array}{cc} -\frac{c_{n}}{2} & \frac{\gamma_{1}a_{1}}{4\omega_{q}}-\frac{\delta +\Delta +k_{2}a_{1}^{2}}{2}\\ \frac{\gamma_{1}a_{1}}{4\omega_{q}}+\frac{\delta +\Delta +k_{2}a_{1}^{2}}{2} & -\frac{c_{n}}{2} \end{array}\right]\;$$

According to Equation (59), the local stability can be analyzed using the trace and determinant of the Jacobian matrix. As $c_{n}$ is always larger than zero here, the critical point can be found while the determinant of the Jacobian matrix is zero. With Det(J)=0, the threshold for $a_{1c}$ is obtained:

(60) $$a_{1c}^{2}=\frac{\frac{\gamma_{1}^{2}}{4\omega_{q}^{2}}-2k_{2}\left(\delta +\Delta \right)-\sqrt{{\left(\frac{\gamma_{1}^{2}}{4\omega_{q}^{2}}-2k_{2}\left(\delta +\Delta \right)\right)}^{2}-4k_{2}^{2}\left({\left(\delta +\Delta \right)}^{2}+c_{n}^{2}\right)}}{2k_{2}^{2}}$$

The threshold implies that: if $a_{1}>a_{1c}$, the transversal vibration may occur, else if $a_{1}<a_{1c}$ there is no transversal vibration. For an $a_{1c}$ in physical meaning, the value in the radical sign must be non-negative. Therefore, the physical condition for the transversal vibration is:

(61) $$\frac{\gamma_{1}^{2}}{4\omega_{q}^{2}}>2k_{2}\left(\delta +\Delta \right)+2k_{2}^{2}\sqrt{{\left(\delta +\Delta \right)}^{2}+c_{n}^{2}}$$

The basic physical condition is also the critical condition for modal coupling vibration. As the amplitude of the longitudinal vibration increases exceeding the critical value $a_{1c}$, the energy transfers from the longitudinal mode to the transversal mode. The stability analysis of the nontrivial solution should be given as the nontrivial solution branches bifurcate. For this parametrically excited system, Near the critical points, the supercritical Hopf bifurcation leads to stable branches, while subcritical Hopf bifurcation leads to unstable branches. To characterize the stability of periodic vibration, Hopf bifurcation of critical points is to be studied. Substituting the critical solution $a_{1c}$ into Equation (39), the following discriminant can be obtained:

(62) $$\Lambda =k_{1}\left(\gamma_{1}^{2}\left(\delta +\Delta \right)+4k_{2}c_{n}^{2}\omega_{q}^{2}\right)$$

If Λ<0, the subcritical Hopf bifurcation occurs, and the jump phenomenon will appear in the transversal mode when the amplitude of the longitudinal vibration exceeds the critical value. On the contrary, if Λ>0, the supercritical Hopf bifurcation occurs, and the longitudinal vibration only induces the small transversal vibration, even the amplitude of the longitudinal vibration is large. For Λ=0, the threshold of amplitude of the longitudinal vibration is minimum, meaning that a relatively smaller electrostatic force could motivate the transversal vibration.

## 7. Dynamic Analysis

To further research the nonlinear dynamical behavior under different Hopf bifurcation parameter range, the influences of electrostatic force and detuning frequency on the system are introduced. In this section, we study the complex dynamical behaviors of electrostatically actuated microbeam using pseudo-arclength continuation method and some interesting phenomena are obtained. The dynamic responses of the system are simulated by Crank-Nicolson method, which is numerically stable.

**Figure 5:**
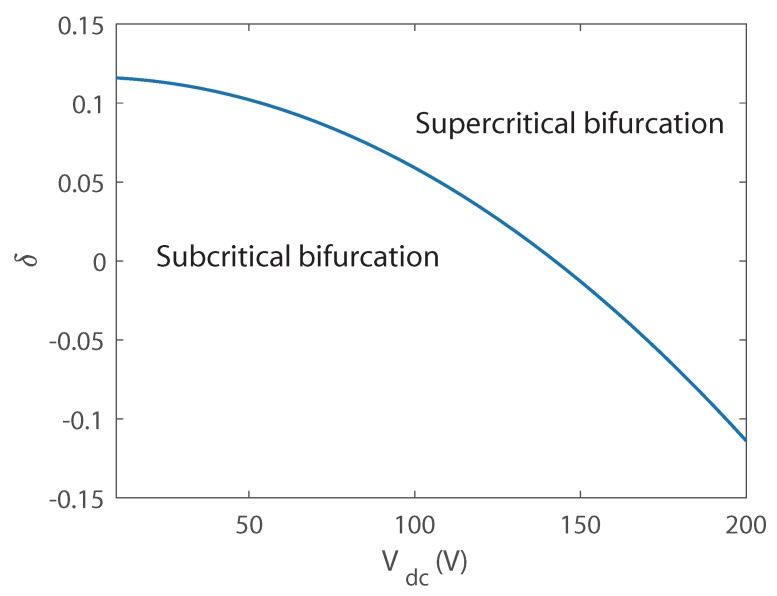
Variation of the bifurcation behavior versus δ and $V_{dc}$.

Firstly, the bifurcation behavior of the longitudinal–transversal coupling vibration is investigated. Figure 5 displays variation of the bifurcation behavior versus $V_{dc}$ and δ. It is found that a larger DC voltage corresponds to a larger δ, indicating that with a larger DC voltage, the modal coupling coefficient is reinforced, and nonlinear modal interactions is enhanced. Here we can explain the phenomenon in Figure 4: in the simulation δ=0.1 is used, with $V_{dc}$ = 30 V, subcritical bifurcation occurs while $V_{dc}$ = 60 V and $V_{dc}$ = 90 V supercritical bifurcation occurs, hence two kinds of force-amplitude responses are obtained.

**Figure 6:**
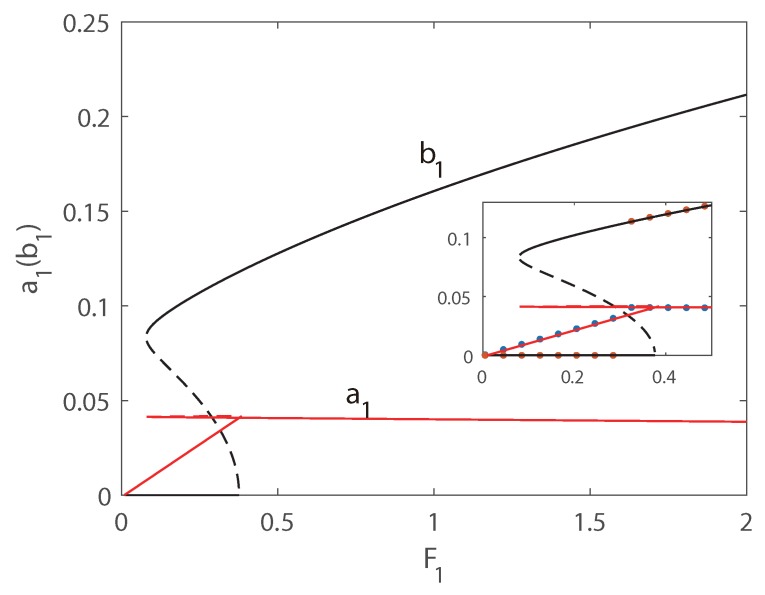
Amplitudes of response as functions of the excitation amplitude $F_{1}$. Line: pseudo-arclength continuation method, Points: long-time-integration method.

**Figure 7:**
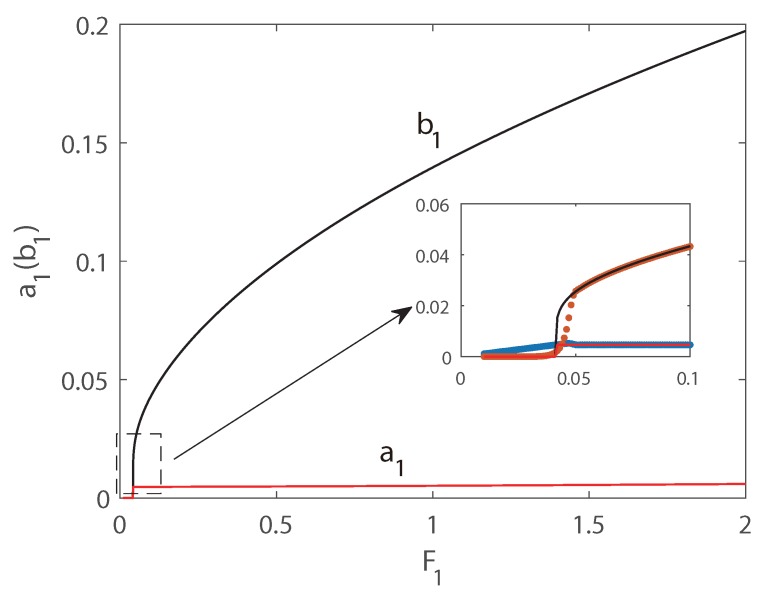
Amplitudes of response as functions of the excitation amplitude $F_{1}$. Line: pseudo-arclength continuation method, Points: long-time-integration method.

In Figure 6 and Figure 7, the first transversal amplitude and the first longitudinal amplitude are plotted as functions of the longitudinal excitation amplitude $F_{1}$. To validate the numerical simulations, both the pseudo-trajectory processing method (line) and long-time-integration method (points) are used in the calculation, showing very good agreement. In the following figures, solid lines always denote stable solutions and dashed lines denote unstable solutions.

In Figure 6, $V_{dc}$ = 140 V and δ = −0.1 is employed, and subcritical Hopf bifurcation occurs. Hence as $F_{1}$ increases, the jump phenomenon appears in the second-order mode and the amplitude of the second mode is much larger than that of the third mode. The phenomenon of saturation can be found for the longitudinal vibration, and the energy transfers from longitudinal mode to transversal mode. In Figure 6, as the external force increases from zero, the amplitude of the longitudinal vibration increases along the natural relationship $A=\frac{F_{1}}{\sqrt{{\left(\Omega^{2}-\omega_{p}^{2}\right)}^{2}+{\left(c_{n}\right)}^{2}}}$, and the amplitude of the transversal vibration still keeps zeros. It is interesting that beyond a critical value of $F_{1}$, the solution of longitudinal vibration loss stability, and another branch of solution dominates the motion. Then the amplitude of the longitudinal vibration becomes independent of the amplitude of the external force, i.e., the saturation occurs. It is interesting that the jump phenomenon occurs in transversal mode, and the jump amplitude is very large. Therefore, taking advantage of the nonlinear features of the frequency response, a large-amplitude jump will provide an effective approach to improve the precision of MEMS switches. In Figure 7, supercritical Hopf bifurcation occurs while $V_{dc}$ = 140 V and δ = 0.1 is adopted. From Figure 7, the phenomenon of saturation of longitudinal mode can also be found. However different from the plots in Figure 6, as the external force increases from zero, the amplitude of the longitudinal vibration increases as well, while the amplitude of the transversal vibration keeps zeros. Beyond a critical value of $F_{1}$, the amplitude of transversal vibration experiences a drastic rise first, and then increases in a slow rate continually.

**Figure 8:**
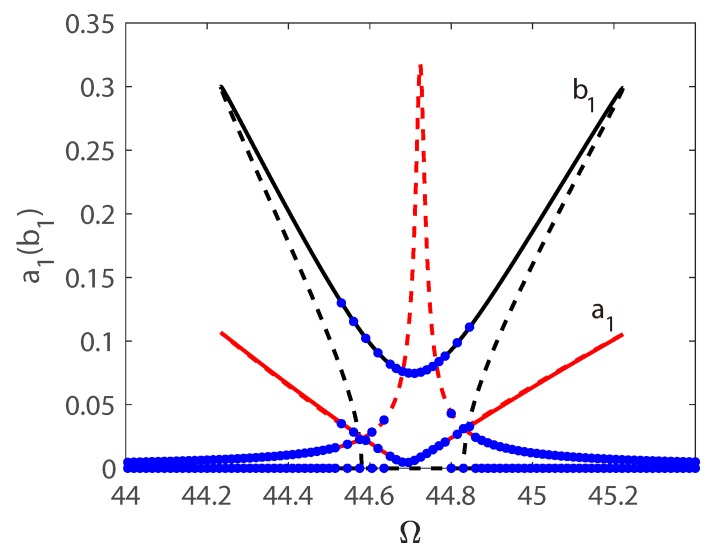
Amplitudes of response as functions of the detuning parameter δ. Line: pseudo-arclength continuation method, Points: long-time-integration method.

Figure 8 shows the amplitude responses of the coupled vibration as function of internal resonance detuning parameter δ. A constant bias voltage $V_{dc}$ = 140 V is applied to supply an electrostatic force. The frequency sweep response shows a slight asymmetry. Before reaching the threshold, the longitudinal vibration operates in its linear regime and the amplitude of longitudinal vibration increases linearly, while there is no transversal vibration. Once reaching the certain threshold, the solution of longitudinal vibration turns to another branch of solution. Meanwhile an amplitude jump of transversal vibration occurs, and the vibration energy transfer from the longitudinal mode into the transversal mode. In the design of switches, the jump phenomenon can be used to change the ON and OFF state, as the amplitude jump is very large, the precision and switch speed can be greatly promoted.

**Figure 9:**
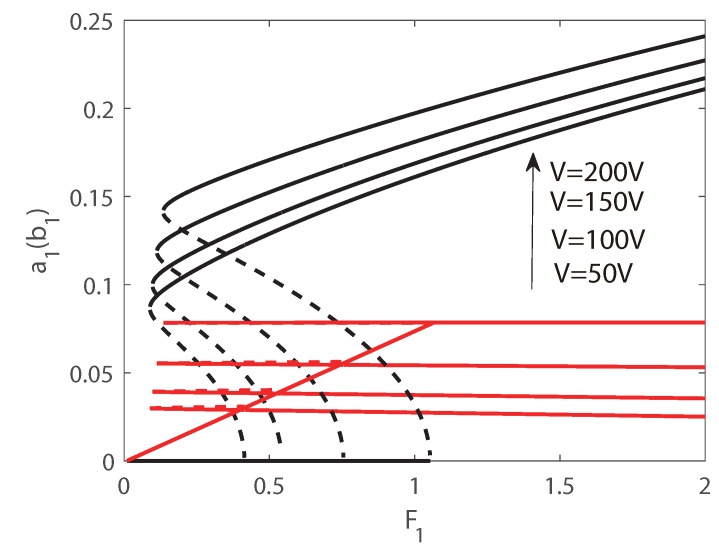
Amplitudes of response as functions of the excitation $F_{1}$ for different $V_{dc}$.

As introduced above, the jump phenomenon is used and the parametric excitation vibration only in the subcritical Hopf regime is taken into consideration in this section. The parametric force-response curves obtained by pseudo-arclength method for different values of DC voltages are plotted in Figure 9. In the calculation, δ = −0.15 is used and $V_{dc}$ ranges from 50 V to 200 V with an interval of 50 V. It is found that with a larger DC voltage, a larger excitation $F_{1}$ is needed to realize the jump phenomenon of the transversal mode. As also seen from the plots, with a larger DC voltage, the amplitude of the jump is larger as well, demonstrating that the performance of the switch can be improved by increasing the DC voltage.

**Figure 10:**
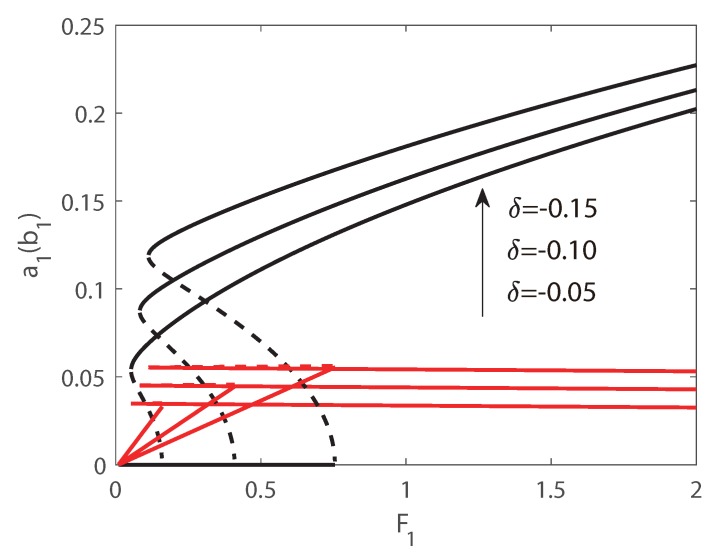
Amplitudes of response as functions of the excitation $F_{1}$ for different δ.

The force-response curves for different detuning parameter δ are plotted in Figure 10. Here $V_{dc}=150$ and δ=(−0.05,−0.1,−0.15) is used. The plots are similar with that in Figure 10. However, the point of the limit point bifurcation and the period-doubling bifurcation are all delayed while δ is smaller, and with a smaller δ the amplitude of the jump is larger. Both in the two figures, a larger saturation value can be found with a larger jump, illustrating that the energy is transferred between the longitudinal–transversal modes interaction.

**Figure 11:**
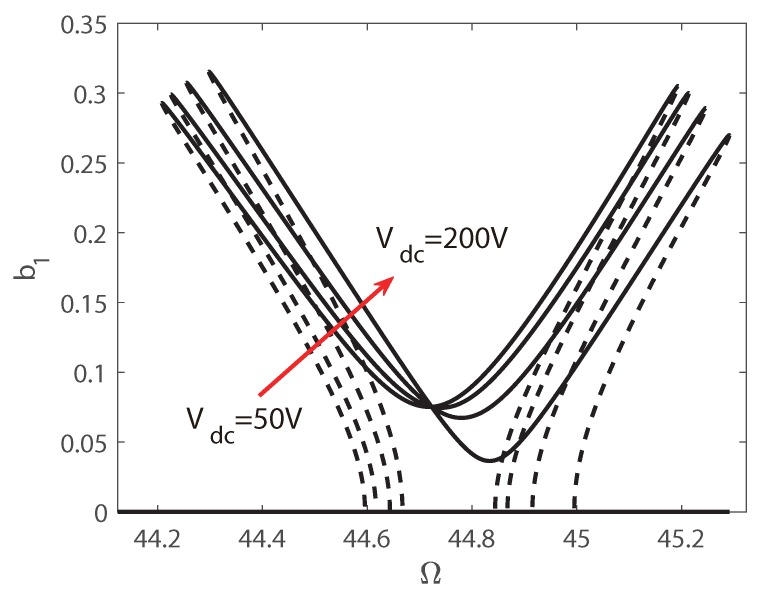
Frequency-response curves for different $V_{dc}$.

Figure 11 shows the frequency sweep response of transversal vibration for different $V_{dc}$. The values of $V_{dc}$ is defined as 50 V, 100 V, 150 V, and 200 V. The whole frequency sweep response curves are similar with the plots in Figure 8. It is visualized that the unsymmetrical configuration can still be found, and the asymmetry is strengthened by a larger DC voltage. Here only the curves for transversal mode are given. The plots show that with a lager bias voltage, the left jump is also bigger while the right jump is smaller. Both the left and right threshold shift right while the bias voltage increases.

## 8. Conclusions

To extend the application of bifurcations of the coupling longitudinal–transversal vibration in the design of MEMS switches, this paper mainly focused on the nonlinear characteristics of an electrostatically actuated microbeam, and the coupled longitudinal–transversal vibration was analyzed in detail. A reduced-order model was obtained by Euler-Bernoulli beam theory and Galerkin’s method, and the obtained single mode Galerkin model was verified by differential quadrature method, demonstrating that the single mode model could describe the dynamic behavior of the system accurately with small deformation assumption. Using the reduced-order model, the physical condition for the energy transfer was obtained by Hopf bifurcation analysis. From theoretical analysis, the critical values of parameters were obtained from the physical condition for the Hopf bifurcation.

The nonlinear behavior of the electrostatically actuated microbeam was analyzed via the pseudo-arclength continuation technique and a direct time integration. The frequency-amplitude responses and force-amplitude responses obtained by the pseudo-arclength continuation technique and a long-time-integration method showed good agreement. From the nonlinear electrodynamical resonance response, the mechanism of energy transfer between longitudinal vibration and transversal vibration was presented. The numerical simulations revealed that the bias voltage, the detuning frequency, and the excitation amplitude of in longitudinal direction played important roles in the capability of MEMS switches. Once the subcritical regime was activated, with a larger bias voltage or a smaller detuning frequency, shifted forced frequency responses were obtained, and the amplitude jump would become larger. The simulations gave a reliable analysis for reasonable design of structural properties, providing a great potential in improving precision of MEMS switches due to the subcritical bifurcation regime.
